# Maturation of the vertebral ring apophysis is delayed in girls with adolescent idiopathic scoliosis compared to the normal population

**DOI:** 10.1007/s43390-024-00908-w

**Published:** 2024-06-07

**Authors:** Lorenzo Costa, Agnes van Lange, Peter R. Seevinck, Winnie Chu, Ludvig Vavruch, Moyo C. Kruyt, René M. Castelein, Tom P. C. Schlosser

**Affiliations:** 1 Department of Orthopedic Surgery, University Medical Center Utrecht, G05.228, 3508 GA, P.O. Box 85500, Utrecht, The Netherlands; 2 Department of Imaging, University Medical Centre Utrecht, Utrecht, The Netherlands; 3 Department of Imaging and Interventional Radiology, Chinese University of Hong Kong at The Prince of Wales Hospital, Sha Tin, China; 4 Department of Biomedical and Clinical Sciences, Linköping University, Linköping, Sweden

**Keywords:** Ring apophysis, Spinal maturation, Adolescent idiopathic scoliosis, Spine development, Secondary ossification

## Abstract

**Purpose:**

The ring apophysis is a secondary ossification center on both sides of each vertebral body, to which the annulus of the intervertebral disc inserts. Recently, its pattern of ossification and fusion to the vertebral body was described for the normal growing spine. The aim of the present study was to investigate the ossification and fusion of the ring apophysis in patients with adolescent idiopathic scoliosis (AIS) and compare it to the normal growing population.

**Methods:**

Ring apophysis maturation along the entire thoracic and lumbar spine was analyzed on CT scans of 99 female, pre-operative AIS patients and compared to 134 CT scans of non-scoliotic girls, aged 12 to 20.

**Results:**

The ring apophysis maturation in AIS patients was delayed at all spinal levels in AIS patients compared to non-scoliotic controls. Ossification starts at T4–T11 at age 12, followed by T1–T5 and L3–S1 at age 15. The fusion process in AIS patients continues longer in the midthoracic region as compared to the other regions and as compared to non-scoliotic controls, with many incomplete fusions still at age 20.

**Conclusion:**

The ring apophysis maturation in AIS is delayed compared to that in the normal population and lasts longer in the mid/low thoracic spine. Delayed maturation of the spine’s most important stabilizer, while the body’s dimensions continue to increase, could be part of the patho-mechanism of AIS.

## Introduction

Adolescent idiopathic scoliosis (AIS) is the most common type of pediatric spinal deformity, typically developing and progressing during pubertal growth [1, 2]. During this time, the increasing loads on the growing spine challenge its delicate rotational stability [3]. The intervertebral disc (IVD) is one of the spine’s most important stabilizers, initially its attachment through the Sharpey’s fibers is into the cartilaginous endplate, that first ossifies and then fuses to the bone of the vertebra, at which stage the spine can be considered ‘mature’ [4–7]. For growth to proceed harmoniously, the developing mechanical properties of the disc must remain in synchronicity with these rapidly increasing loads [3]. In case of a mismatch between increasing asymmetrical spinal loading, and mechanical maturation of the IVD, spinal deformity may develop [3, 8–11].

Because of the timing of onset and progression of AIS, it is hypothesized that in AIS patients the intervertebral discs mature slower compared to non-scoliotic adolescents and asynchronous with the increase in spinal loading. Recently it has been shown that, utilizing high-resolution CT scans, it is possible to quantify the ossification and fusion of the ring apophyses along the thoracic and lumbar scoliotic spine. Hence, the objective of this study was to investigate the ossification and fusion of the ring apophysis in patients with AIS and compare it to the normal growing population.

## Methods

### Study population

For this retrospective cohort study, high-resolution whole spine computed tomography (CT) scans of patients with an indication for AIS surgery, aged 10 to 21 years were included. The CTs were obtained for navigation purposes and obtained from two academic hospitals [12]. Inclusion criteria were: female patients between 10 and 21 years of age, presence of idiopathic scoliosis, high-resolution images of the spine from T1 to L5 available (slice thickness ≤ 1 mm and slice interval ≤ 1.5 mm). Exclusion criteria were presence of spinal pathology other than AIS, previous surgery, bone disorders syndromes associated with growth disorders, growth hormone treatment and insufficient CT-scan quality including movement artifacts. According to all available information, subjects were representative for scoliotic adolescents with need of surgical treatment as stated by the SRS and SOSORT criteria [13, 14]. Age, gender, Lenke classification, magnitude of the primary and compensatory curves, the apex level and the Cobb end vertebrae were documented. For a control group, data from a prior study involving high-resolution CT scans of 134 Caucasian females aged 12–20 without spinal pathology were used [15].

### Image analysis

CT-scans were analyzed by two trained observers. Multiplanar images of the exact mid-sagittal and mid-coronal plane of each individual vertebra from T1 to L5 were reconstructed to visualize the true anterior, posterior, and lateral parts of each endplate/ring apophysis, using *Scoliosis Analysis 7.2 (Image Sciences Institute, UMC Utrecht, the Netherlands)*. Moreover, the observers scrolled three slides forward and three backward from the mid-sagittal and mid-coronal planes to confirm accurately visualize the rings.

To classify the ossification and fusion process of the ring apophysis, a previously developed classification system was used [15]. In AIS, however, due to compression/shortening on the concave side of the scoliotic curve, distinguishing the ring from the cortical corner of the body can be difficult. Therefore, if for one of the four sides of the ring apophysis it was not possible to differentiate phase 0 (no ossification) to phase 2 or 3 (complete fusion), the other three visible regions were used (Table 1).

**Table 1:** After defining the phase of the ring apophysis maturation in the anterior, posterior, left and right region of interest for each ring, the ring apophysis maturation scale was determined [12]

Step 1	Phase 0	Phase 1	Phase 2	Phase 3	
Phasing process	No ossification, no fusion	Ossification, no fusion	Incomplete fusion	Complete fusion	

Step 2	ROI = region of interest	ROI = region of interest	ROI = region of interest	ROI = region of interest	ROI = region of interest
Ring apophysis maturation scale	Phase 0 in all ROI	Phase 1 in 1–3 ROI	Phase 1 in all ROI	Phase 2 in 1–4 ROI and/or phase 3 in 1–3 ROI	Phase 3 in all ROI

ROI = region of interest

### Data analysis

Statistical analysis was performed using IBM SPSS Statistics for Windows, Version 29.0.0.0 (241) Armonk, NY: IBM Corp. Mode and ranges were calculated for ordinal data on curve characteristics. Mean and standard deviation (SD) were computed for both main and compensatory curve Cobb angles. Median, mode and interquartile (IQR) range of the maturation stages were calculated for each age group and spinal level. Percentages were computed for each subject to determine the visibility of the regions of interest. The relation between median ring apophysis maturation stage and age was analyzed with Spearman’s rank correlation coefficient. Ring apophysis maturation stages at the different ages were compared between the control and the AIS group using a Chi-square test. A p-value was considered statistically significant if < 0.05.

## Results

### Study population

99 AIS patients and 134 controls could be included in this study, corresponding to 3366 and 4556 ring apophyses, 83% was Asian, 17% Caucasian. As no patients were 10, 11 or 21 years old, the analysis started from the age of 12 years and ended at the age of 20 with a mean age of 16. Most subjects (41%) had Lenke type 1 curves. The apical area was mainly located at T8 (range T5–T12) for the main curve and at L3 for compensatory curves (range L1–L4). Mean Cobb angles for the main and the compensatory curves were, respectively, 54^°^ (SD = 12) and 36^°^ (SD = 13).

### Ring apophysis maturation and comparison with the normal population

All four sides of the T1–L5 ring apophyses were fully visible in all controls and in 1720 (51%) of the 3366 apophyses in scoliotic patients, three sides were visible in 1472 (44%) of the apophyses in scoliosis patients. There were differences in visibility between the concave and convex sides and apical and non-apical regions. In the primary curves, 86% of the convex ROI of the ring was visible, while only 21% of the concave ROIs were visible. In the peri-apical area, considered as the apical vertebra and one vertebra above and one below, the concave ROI of the rings was visible in only 2%.

**Fig. 1:**
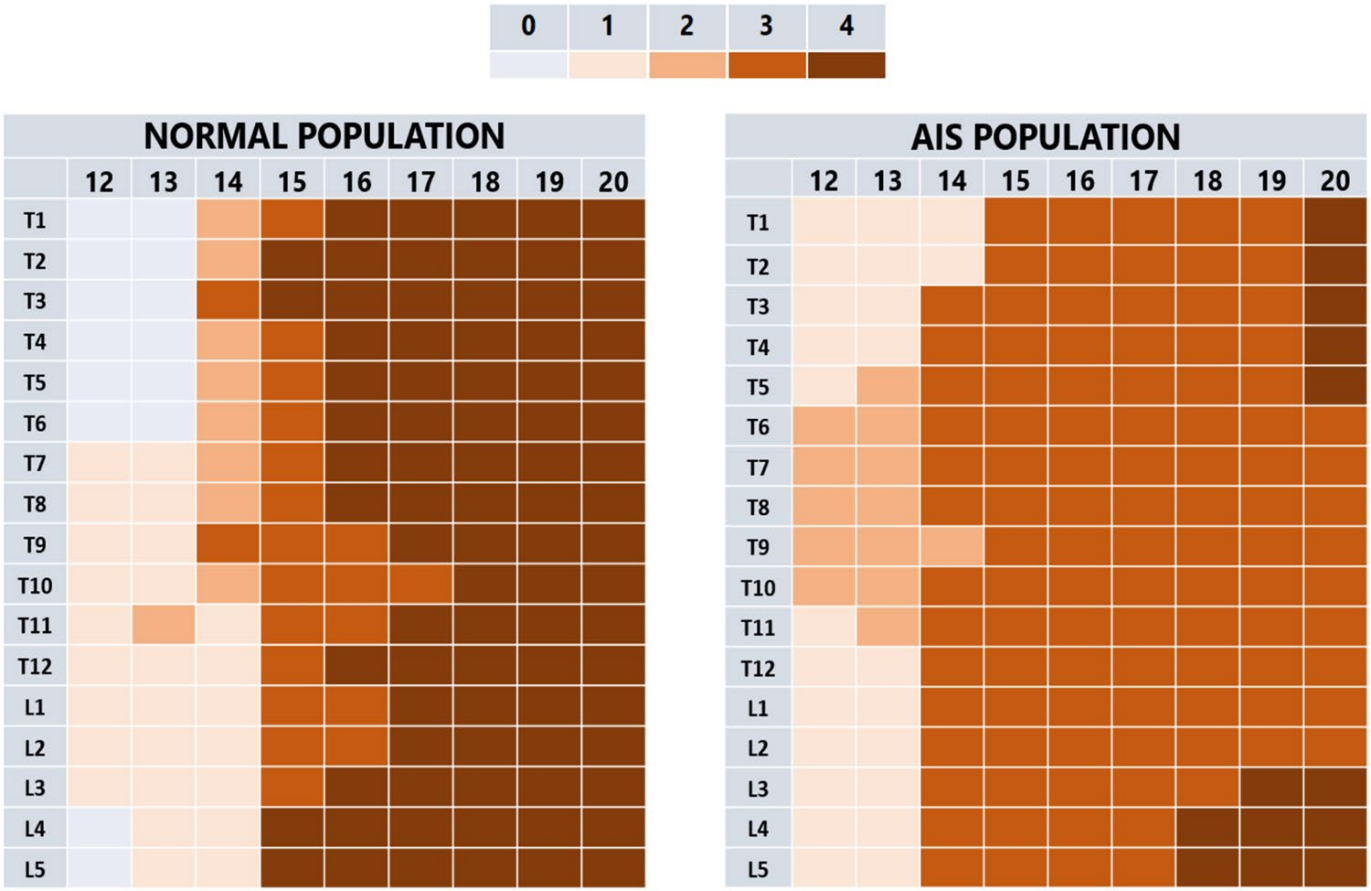
The mode of maturation of the apophyseal rings in the different thoracic and lumbar spinal levels, for each age groups and between the normal and the AIS population. The legend shows the ring apophysis maturation stage [12]

**Fig. 2:**
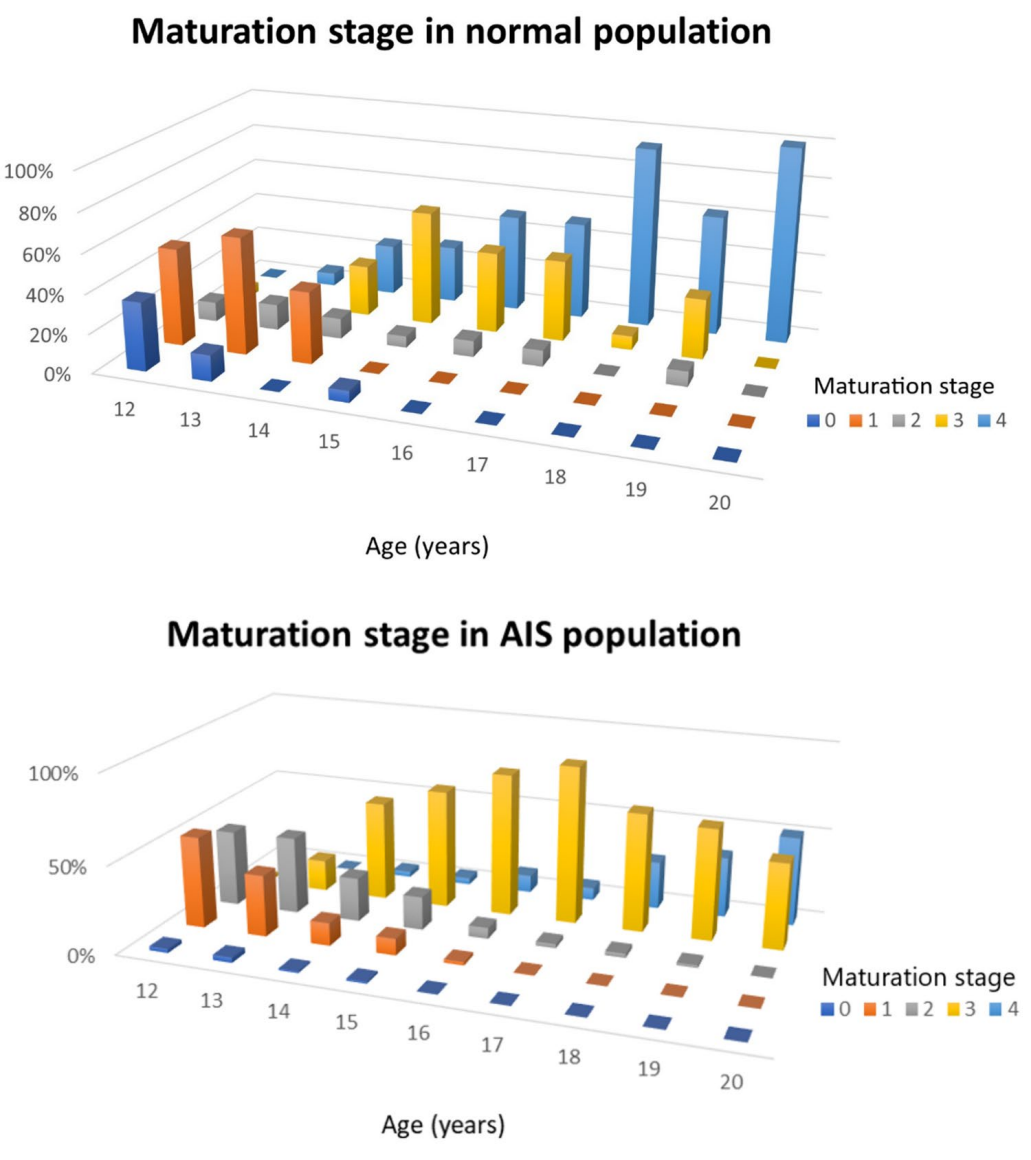
The modes of maturation stage of the ring apophyses are shown for the normal and AIS population. The colors and numbers on the z axis represent the ring apophysis maturation stage. X axis represents the age groups and the y axis the proportion of patients in this stage

**Fig. 3:**
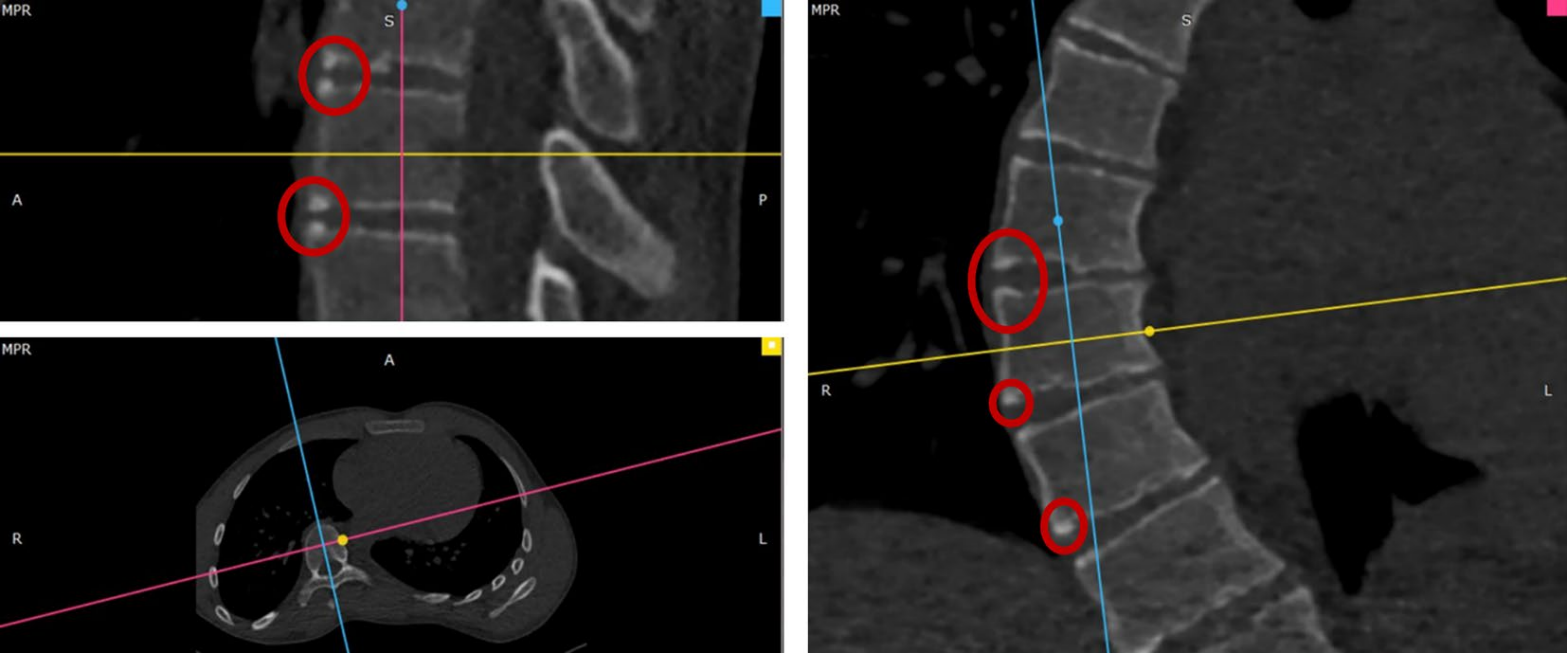
Representation of the ring apophysis in an 18-year-old female with AIS, Lenke 1, T5–T12 (apex at T8) and 56 degrees coronal Cobb angle. Maturation scale of the superior and inferior ring apophysis on the anterior and right side were 3, representing incomplete maturation

Maturation of the ring apophysis is a process that varies for each age and for different spinal regions. The maturation stage in AIS female patients significantly correlated with age (R = 0.90, *p* < 0.001). The maturation stage and IQR range slowly increased per age group as shown in Table 2, and this maturation process differed between spinal levels: while ossification occurred earlier from T4 to T11, the fusion process appeared to be delayed in the mid and low thoracic spine. This pattern can also be found in the normal population in a similar manner. However, fusion of the apophysis to the bone of the vertebral body occurs earlier in non-scoliotic girls as compared to the AIS population, with still many incomplete fusions at age 20, as shown in Figs. 1, 2 and 3 (*P* < 0.001).

**Table 2:** The This table represents the mode, median and interquartile range (IQR) of rings’ maturation during the ages

Age (n)	Mode ring apophysis maturation stage (IQR range; median)
12 (10)	1(1–2; 1)
13 (13)	2(1–2; 2)
14 (12)	3(2–3; 3)
15 (13)	3(2–3; 3)
16 (17)	3(3; 3)
17 (10)	3(3; 3)
18 (11)	3(3–4; 3)
19 (8)	3(3–4; 3)
20 (5)	4(3–4; 3)

The n°: represents the number of images included in that age group

**Fig. 4:**
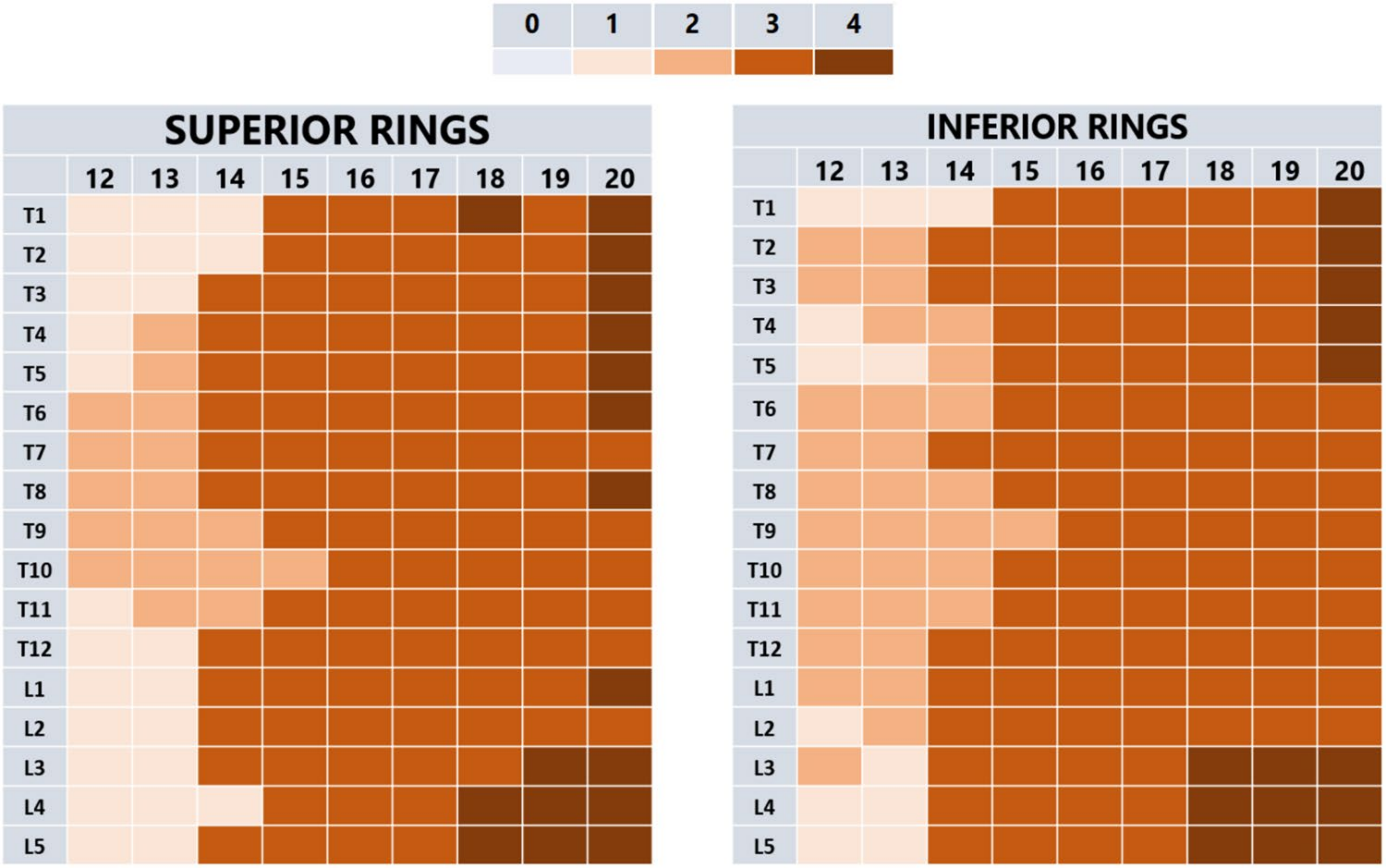
The difference in mode maturation in AIS of the apophyseal rings in the different sections of the spine between the age groups divided between superior rings and inferior rings. The legend shows the ring apophysis maturation stage [12]

**Fig. 5:**
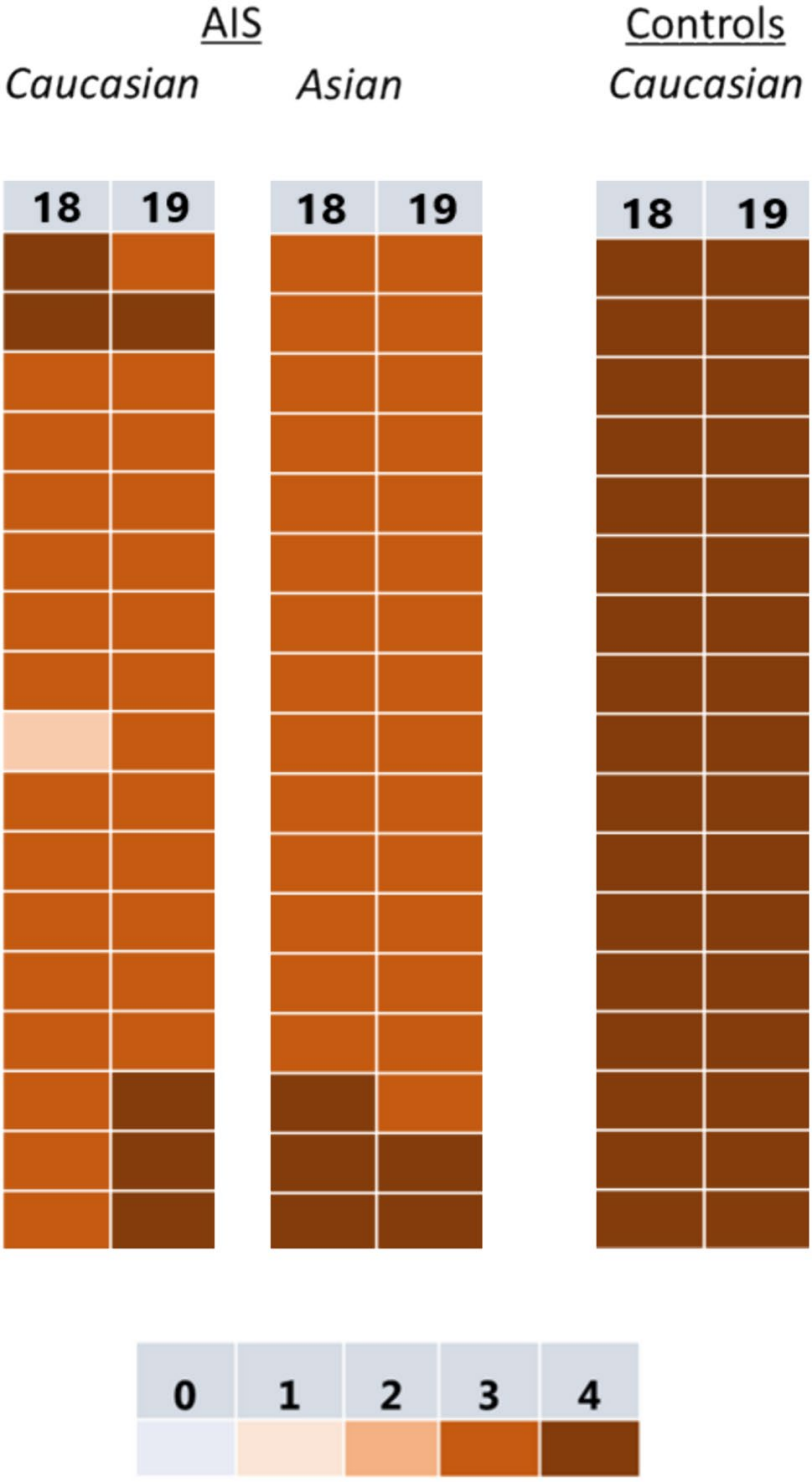
Illustration on the ring apophysis maturation stage at 18 and 19 years old in the Asian and Caucasian AIS patients and the Caucasian control cohort

No specific differences could be detected between the ossification and fusion in the superior *versus* the inferior ring apophyses (*P* = 0.206), or between patients with a Caucasian or Asian background (*P* = 0.130) (Figs. 4 and 5).

## Discussion

The human growing spine is a mosaic of growth plates, with three primary ossification centers ossifying before birth, the neurocentral cartilage closing before age 10, and secondary ossification centers at the top and bottom of the vertebral bodies and tips of the transverse and spinous processes that eventually fuse during the second or third decade of life [15–18]. The maturation of the peripheral portions of the epiphysis on both the cranial and caudal side of each vertebral body, the ring ‘apophysis’, reflects the maturation of one of the human spine’s most important stabilizer because of insertion of the anchoring fibers of the annulus of the intervertebral disc, the Sharpey fibers [7, 9, 19, 20].

In the current study, we compare high-resolution spinal CTs of 99 female patients with surgical range AIS to 134 CTs of non-scoliotic adolescent and young adult females [21–23]. None of the CTs were obtained for the purpose if this study, those of scoliotic patients were obtained for navigation purposes, those of the controls for various reasons such as poly-trauma or infectious disease. The pattern of the ossification and fusion process of the ring apophysis in AIS was similar to the normal growing female spine: the mid/low thoracic region (T4–T11) ossifies first and later the upper thoracic (T1–T5) and lower lumbar region (L3–S1). Similarly, the fusion process lasts longest in the mid/low thoracic region as compared to the other regions. These findings are consistent, although at a different age, with the sequence of closure of the neurocentral junctions in the normal young growing population [15, 24]. However, the overall maturation process of the ring apophyses appeared to be some years delayed in the AIS population as compared to the normal population. Specifically, in the low thoracic and thoracolumbar region, fusion of the ring apophysis to the vertebral body occurs from age 18 and lasts beyond age 20 in AIS, as compared to the starting age of 15 and almost full fusion at age 20 in non-scoliotic females.

This phenomenon was found both on the convexity and on the concavity, suggesting that it may be independent of the developing curve. If the disc indeed remains ‘infantile’ while the body dimensions are reaching the ‘adult’ stage, these maturation phenomena are not in harmony, which may result in a relative overloading of the intervertebral disc–vertebral body complex. This mismatch between a maturing ligamentous insertion and increasing forces is also known for instance in the tibial tubercle, where forces that approach the ‘adult’ stage on the still immature insertion of the patellar tendon lead to Osgood–Schlatter disease. The question of course is whether this delayed maturation of the disc is part of a more general delay in maturation in scoliotic girls. This does not seem to be the case as Modi et al. reported no differences in Risser staging and digital skeletal age on hand radiographs between scoliotic and non-scoliotic patients, Sanders et al. report Tanner–Whitehouse-III and Greulich and Pyle references similar to what can be expected for the normal population. Based on general anthropometrics, Nissinen et al. showed that scoliotic girls even seem to have an earlier instead of a later PHV and peak sitting height velocity [25–27]. Therefore, this apparent mismatch between maturation of the disc–vertebral body complex and the rest of the body seems to be scoliosis specific and need further investigation.

A limitation is that the observed delay may be biased for several reasons. First, the observed delay could be a result of asymmetrical loading of the IVDs and ring apophyses [28]. This possibility however is largely denied because delayed maturation was found in the entire spine and there was no difference between concave and convex vertebral maturation. However, similar to the findings of Makino et al., the ring apophysis was often not detected on the concave side around the apex of the scoliotic curve [29]. Second, only data of operative AIS patients were included. It is possible that the indication for surgery skewed the data as these patients are the ones with the more severe curves. However, it seems very unlikely that a subjective, man-made surgical limit would play a role in a maturation process. Furthermore, the majority of the AIS group consisted of Asian girls that were not represented in the control group. However, there are no indications that Asian girls mature later and both, the Caucasian and Asian AIS patients in our dataset had incomplete fusions at age 18–20 (Fig. 5) [30]. Finally, the multicenter study population did not permit extensive comparison between the different age groups or ethnicities, as not all age cohorts did have more than 10 patients included.

## Conclusion

Analysis of the ossification and fusion process of the ring apophysis in AIS and non-scoliotic adolescent or young adult females, demonstrates that the ring apophysis maturation in AIS is delayed compared to that in the normal population and that this maturation lasts longest in the mid/low thoracic spine. Delayed maturation of the spine’s most important stabilizer, while the body’s dimensions continue to increase, could be part of the patho-mechanism of AIS. Given the fact that this difference occurs throughout the spine and is irrespective of concavity or convexity of the curve, it seems likely that this phenomenon is not secondary to the scoliotic curve but possibly a primary delay in maturation of the vulnerable regions of the scoliotic spine.

## Data Availability

The data presented in this study are available on request from the corresponding author. The data are not publicly available due to ethical and privacy reasons.
